# ERASE: a feasible early warning tool for elder abuse, developed for use in the Dutch emergency department

**DOI:** 10.1186/s12873-024-00971-6

**Published:** 2024-04-03

**Authors:** Miriam E. van Houten, Lilian C. M. Vloet, Marcel G. M. Olde Rikkert, Brigitte van de Kerkhof-van Bon, Anneriek de Rooij, Marieke Verhoeven, Wilhelmina M. E. Bil, Jacinta A. Lucke, Yvonne Schoon, Sivera A. A. Berben

**Affiliations:** 1 Department of Geriatric Medicine, Radboud University Medical Centre, PO Box 9101, 6500 HB Nijmegen, The Netherlands; 2 Research Department of Emergency and Critical Care, School of Health Studies, Knowledge Centre of Sustainable Healthcare, HAN University of Applied Sciences, PO Box 6960, 6503 GL Nijmegen, The Netherlands; 3 Trompetter & Partners Social Medical Expertise, Utrechtseweg 75, 3702 AA Zeist, The Netherlands; 4 Radboud University Medical Centre, Radboud Institute for Health Sciences IQ Healthcare, P.O. Box 9101, 114, 6500 HB Nijmegen, the Netherlands; 5 Radboudumc Alzheimer Centre, Donders Insititute of Medical Neuroscience, Department of Geriatrics, PO Box 9101, 6500 HB Nijmegen, The Netherlands; 6 Canisius Wilhelmina Hospital, 6532 SZ Nijmegen, The Netherlands; 7 Jeroen Bosch Hospital, 5223 GZ ’s Hertogenbosch, The Netherlands; 8 Department of Geriatrics, OLVG, Amsterdam, 1061 AE The Netherlands; 9 Spaarne Hospital, 2035 RC Haarlem, The Netherlands; 10 Department of Geriatrics, Radboud Institute for Health Sciences, Radboud University Medical Center, PO Box 9101, 114, 6500 HB Nijmegen, The Netherlands

**Keywords:** Elder abuse, Warning tool, Feasibility, Emergency department

## Abstract

**Background:**

Elder abuse is a worldwide problem with serious consequences for individuals and society. To effectively deal with elder abuse, a timely identification of signals as well as a systematic approach towards (suspected) elder abuse is necessary. This study aimed to develop and test the acceptability and appropriateness of ERASE (EldeR AbuSE) in the emergency department (ED) setting. ERASE is an early warning tool for elder abuse self-administered by the healthcare professional in patients ≥ 70 years.

**Methods:**

A systematic literature review was previously conducted to identify potential available instruments on elder abuse for use in the ED. Furthermore, a field consultation in Dutch hospitals was performed to identify practice tools and potential questions on the recognition of elder abuse that were available in clinical practice. Based on this input, in three subsequent rounds the ERASE tool was developed. The ERASE tool was tested in a pilot feasibility study in healthcare professionals (*n* = 28) working in the ED in three Dutch hospitals. A semi-structured online questionnaire was used to determine acceptability and appropriateness of the ERASE tool.

**Results:**

The systematic literature review revealed seven screening instruments developed for use in the hospital and/or ED setting. In total *n* = 32 (44%) hospitals responded to the field search. No suitable and validated instruments for the detection of elder abuse in the ED were identified. The ERASE tool was developed, with a gut feeling awareness question, that encompassed all forms of elder abuse as starting question. Subsequently six signalling questions were developed to collect information on observed signs and symptoms of elder abuse and neglect. The pilot study showed that the ERASE tool raised the recognition of healthcare professionals for elder abuse. The tool was evaluated acceptable and appropriate for use in the ED setting.

**Conclusions:**

ERASE as early warning tool is guided by an initial gut feeling awareness question and six signalling questions. The ERASE tool raised the recognition of healthcare professionals for elder abuse, and was feasible to use in the ED setting. The next step will be to investigate the reliability and validity of the ERASE early warning tool.

**Supplementary Information:**

The online version contains supplementary material available at 10.1186/s12873-024-00971-6.

## Background

Elder abuse is a worldwide problem with serious consequences for individuals and society. It is associated with increased psychological stress, morbidity and mortality and increased use of healthcare resources, especially emergency services [1, 2]. Elder abuse is defined as a single, or repeated act, or lack of appropriate action, occurring within any relationship where there is an expectation of trust, which causes harm or distress to an older person. [3]. It can take on various forms, such as financial, physical, psychological and sexual abuse, and can be the result of (intentional or unintentional) neglect [3]. A systematic review [4] estimated a globally pooled prevalence rate for overall elder abuse in the community at 15.7% in people 60 years and older. The included studies were geographically diverse (28 countries). In the Netherlands one in twenty community-dwelling people aged 65 and over experienced elder abuse (from the age of 65 or later) and one in fifty experienced elder abuse on an annual basis [5]. Recently some studies have reported an increase in rates of elder abuse during the COVID-19 pandemic, possibly due to the isolation and social distancing measures, among other factors [6–8]. Recognition of elder abuse by healthcare professionals is complex, due to the deficit in the level of awareness and knowledge on elder abuse among healthcare professionals [9, 10]. Furthermore, when not properly trained, healthcare professionals find it difficult to address the issue to the victim [11]. Therefore, there is a strong need for education and specific training on the recognition of elder abuse [9, 10, 12].

To effectively deal with elder abuse, a timely identification of signals, as well as a systematic approach towards cases of (suspected) elder abuse, is necessary. Several screening instruments and tools have been developed to detect possible signs of elder abuse. These instruments are not always suitable for use in the emergency department (ED) setting [13], which is unfortunate, because the ED-visit is an important opportunity to detect cases of elder abuse [14]. The Dutch guideline on elder abuse recommended that healthcare professionals working in the ED should at least develop an awareness for the detection of elder abuse. It was stated that this specific awareness could be supported by asking themselves the following gut feeling question (pertaining to all individuals aged 70 and over): “Do you have a feeling or suspicion of elder abuse?” [15]. Therefore the aim of this study was to develop and test the acceptability and appropriateness of an early warning tool for elder abuse, called ERASE (EldeR AbuSE), in which a gut feeling question is incorporated.

## Methods

### Development of ERASE

#### Study design

The ERASE early warning tool was developed between November 2019 and August 2020. It is intended for all professionals in the ED. The development of the ERASE warning tool had a multimethod design, including a systematic literature review, a field-consultation and an acceptability and appropriateness assessment.

### Study setting and population

The target population for the development of the early warning tool encompassed all patients aged 70 years and older that were admitted to the ED. Furthermore, the acceptability and appropriateness of the ERASE tool in clinical practice were tested in physicians, medical specialists, (specialized) nurses and nurse specialists working in the ED setting in three Dutch (general teaching) hospitals (Hospital H1, H2 and H3). The hospitals were located in the East, South and North of the Netherlands. All had a geriatric department and multiple geriatricians working 24/7 h a week.

### Study protocol-development process

Input for the development process was a systematic literature review and a field-consultation in Dutch hospitals to identify existing screening instruments/tools and best practices on elder abuse used in the ED setting. For the systematic literature review, the aim was to include instruments and tools that were brief and compact, suitable for use in the ED setting, valid in detecting all types of elder abuse and self-administered by the healthcare professional without direct questioning of the patient him/herself. Medline, Embase and Cinahl databases were searched from 2005-May 2019 (see search strategy in Additional file 1). We included articles describing the validity and/or reliability of screening instruments to identify elder abuse in healthcare. Articles specifically aimed at instruments developed for the nursing home setting were excluded. All articles were screened on title and abstract by two independent reviewers (SB, MVH). In case of doubt, a third reviewer (LV) was asked to make a final decision. In addition, reference lists and citing of included articles were screened (SB, MVH) and potentially relevant new publications were screened in a similar way. Screening instruments/tools developed for use in the hospital/ED setting, or instruments/tools where the clinical setting of healthcare setting was not further specified, were included in the analysis.

The field consultation was conducted among local officers or healthcare professionals on domestic violence and elder abuse in Dutch hospitals. The researchers made an inventory of which instruments/tools for early identification of elder abuse are currently used in these hospitals, and identified best practices. The respondents of the field consultation were approached by e-mail or telephone via various routes: National Association of local officers for Child Abuse and Domestic Violence (LVAK), National Platform Combatting Elder Abuse (LPBO), Dutch Association for Emergency Care Nurses (NVSVH), Dutch Association for Emergency Medicine Physicians (NVSHA) and the National HIX (HIX = electronic patient file) domestic violence user group. In total, 73 Dutch hospitals were approached to identify which tools, instruments and/or best practices for screening elder abuse were used in their organization. We collected information on the content and number of items the instrument/tool consisted of, the time of administration, the target group and professional who should administer the instrument/tool, the psychometrics of the instrument/tool, the presence of a local officer on domestic violence/elder abuse and whether there was a multidisciplinary team meeting on elder abuse held (regularly) in the hospital.

Based on the systematic literature review, guideline and field consultation, the first concept version of the ERASE tool was constructed by a multidisciplinary development group. This group consisted of experts (e.g. two senior researchers, a geriatric nurse and a geriatric nurse specialist, three officers on domestic violence and elder abuse, a geriatrician and two emergency physicians). In total, 14 multidisciplinary meetings were organized in the development process.

Criteria for the development of the tool were: the instrument should be short and easy to use in clinical practice (max. 5 min to fill in), the tool should cover a gut feeling question as a prescreener (based on the recommendation in the Dutch guideline on elder abuse) [15], followed by a maximum of six additional signalling questions. The target group professionals for the tool were physicians and nurses, with an initial basic training on the recognition of elder abuse, working in the ED.

The concept version of the ERASE tool was constructed in iterative rounds. First, the researchers provided an overview of the literature with the different tools to detect elder abuse, including their variables or elements, psychometric qualities, application in clinical practice such as patients and setting, duration of the assessment and needed professional skills and knowledge. Second, an overview with the same variables of existing practice based instruments was provided, based on the field consultation. However, in the field consultation we found no psychometric properties, as these instruments were not validated.

We started with formulating the one single starting question for the screening on elder abuse and neglect. The content of this question needed to cover all areas of elder abuse and neglect. In three iterative rounds this single starting question was modified. In the first round the question was formulated in the multidisciplinary group discussion, in the second round potential missing aspects could be added and prioritized, and in the third round the question was further modified. The experts in the development group were invited to provide (written) feedback before, during and after the expert meetings. The researchers collected this input for the discussion in the next round. Decisions were made by consensus. This iterative process was also used for the development of the signalling questions and the corresponding pop-up examples.

For the signalling questions, variables and elements of the various instruments were categorized, for instance all questions on the recognition of physical abuse were summarized. Examples of categorized variables (such as physical abuse) were prioritized in the group meeting, based on the level of evidence and the clinical relevance for the context of the ED. Furthermore, the first draft of this signalling question was developed e.g. to detect observed signs of physical abuse. This was followed by a second and third iterative round. Examples that initially were not included in the signalling questions, were used for the corresponding pop-ups. Also here priorities were set, on the one hand to provide a broad support for health professionals to recognize various types of elder abuse, and on the other hand to keep the early warning tool short to use.

### Study protocol-acceptability and appropriateness of the ERASE tool

In each of the three hospitals ten healthcare professionals (respondents) were asked to use the ERASE tool to screen on elder abuse, each in eight older persons in the order of arrival, aiming for in total 240 measurements with the ERASE tool. A (pre-pandemic) estimate of a prevalence of 2% elder abuse was chosen based on a systematic review of literature in the Dutch Guideline on elder abuse [15] and a Dutch prevalence study of elder abuse among community dwelling older persons [16]. Professionals were purposefully recruited and informed by the local members of the development group team, with a minimum of three and a maximum of five physician respondents per hospital. Professionals (respondents) were informed by a standard PowerPoint presentation on the pilot feasibility study, the ERASE tool and the informed consent procedure. They received no additional training regarding the recognition of elder abuse. The basic training was part of the local hospital policy and consisted of an e-learning provided by an external party, focusing on basic knowledge on elder abuse/domestic abuse and child abuse, ethics and law. Criteria for the inclusion were: 1. working at an ED; 2. availability in the pilot period; 3. willingness to test the ERASE tool; 4. completion of a basic training on the recognition of elder abuse; 5. willingness to provide feedback by filling in the questionnaire.

The acceptability and appropriateness of the ERASE tool was tested by a semi structured online questionnaire. Acceptability reflects the perception of the healthcare professional whether the questions and the format of the ERASE tool regarding to content, complexity, comfort, delivery and credibility are agreeable, palatable, or satisfactory for the professionals. [17]. Appropriateness reflects the perceived fit, relevance, or compatibility of the ERASE tool for the practice setting and/or perceived fit of the tool to address elder abuse [18]. The questionnaire consisted of closed questions pertaining to demographic variables of the professionals who participated in the pilot test (profession, age and gender of the respondents) and closed questions on the acceptability and appropriateness of the ERASE tool with answering options on a five-point Likert-style scale or dichotomous answering options (yes/no). Additional free text options were also provided in order to enrich the feedback process. See Additional file 2.

### Data collection and statistical analysis online questionnaire

The researchers sent a hyperlink of the online questionnaire in Form Desk (Innovero Software Solutions B.V.) to the members of the development group in each hospital. Subsequently, the latter provided the link to all the respondents in their hospital, to ensure the privacy of the respondents. The data were exported to an Excel file (Excel software version 2018). Quantitative data were analyzed with descriptive statistics (count and percent, mean and standard deviation). The free text data of additional remark boxes were qualitatively content analysed by two researchers (MvH, SB) independently. As it concerned additional data aimed to detect feedback or suggestions for improvement of the tool, or potential barriers and facilitators for implementation, we did not use specific qualitative research software or coding trees.

### Ethics approval and consent to participate

Potential respondents (healthcare professionals) received written information on the purpose of the study, data management, privacy aspects, and the required time investment according to the Medical Research with Human Subjects Law. After providing the study details, the professionals were asked to provide informed consent following the declaration of Helsinki. Due to the data collection during the Covid-19 period and potentially highly entrusted staff, we asked respondents for oral informed consent. Because no patients but only healthcare professionals were involved in the assessment of acceptability and appropriateness of the tool, and because the questionnaire did not pose any additional (mental) risk for these professionals, no formal approval of the ethics commission was deemed necessary according to the Medical Research with Human Subjects Law, and therefore approval was waived by the research group.

## Results

### Development of ERASE

#### Systematic literature review

**Fig. 1:**
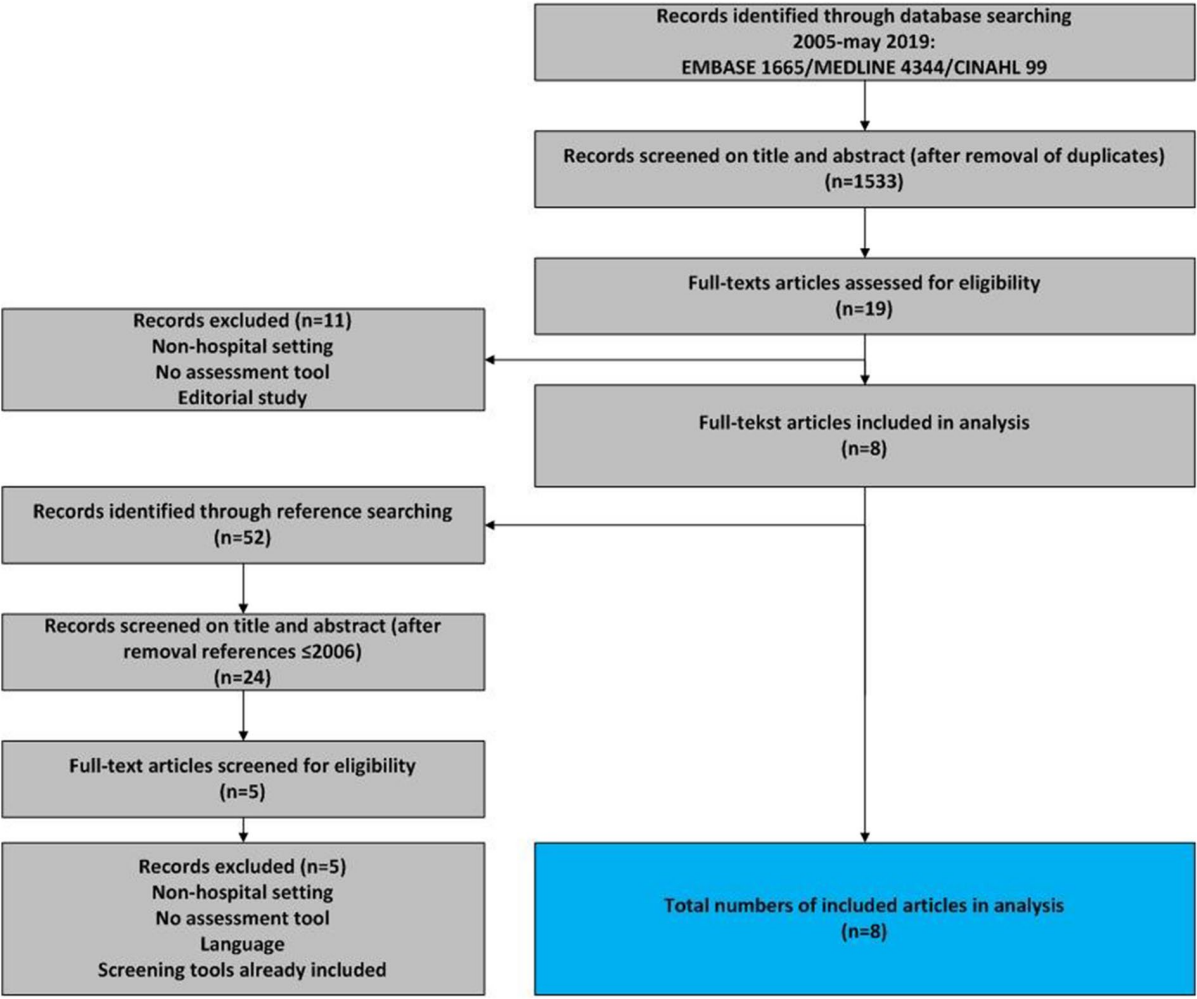
Study selection process

In total, 6108 literature records were identified through database searching (see Fig. 1 for study selection process).

After screening on abstract, full text and reference searching, a total of eight studies were included in the analysis, describing thirty-six screening instruments [13, 14, 19–24]. Seven of the thirty-six instruments were developed for the hospital and/or ED setting: 1. the Elder Assessment Instrument (EAI) and 2. The Expanded Indicators of Abuse (E-IOA) [25, 26]; 3. the Emergency Department Senior Abuse Identification tool (ED Senior AID tool) [14]; 4. the Index of spouse abuse-physical (ISA-P) [20]; 5. the partner violence screen (PVS) [20]; 6. the Two questions abuse screen (TQAS) [20] and 7. the Clinical Signs of Neglect Scale (CSNS) [22]. One instrument was specifically designed for the setting of a dialysis department [23]. In three instruments the healthcare or clinical setting was not further specified: 1. the Conflict Tactics Scale (CTS) [24]; 2. the Health, Attitudes Toward Aging, Living Arrangements and Finances assessment (HALF) [20]; and 3. the American Medical Association screen for various types of abuse and neglect (AMA-STVAN [20]; See Table 1.

**Table 1:** Overview screening tools ED/hospital setting/clinical setting not specified

Tool	E-IOA ^(1)^ Cohen, 2006	EAI ^(2)^ Fulmer, 1984 & 2000	ED senior AID tool ^(3)^ Platts-Mills, 2018	CTS ^(4)^ Strauss, 1979	HALF ^(5)^ Ferguson & Beck, 1983	ISA-P ^(6)^ Hudson&Mcintos, 1982	PVS ^(7)^ Feldhaus, 1997	TQAS ^(8)^ McFarlane, Greenberg, Weltge & Watson, 1995	Questionnaire hemodialysis ^(9)^ Mahmoudian, 2018	CSNS ^(10)^ Friedman, 2017	AMA-STVAN ^(11)^ AMA, 1992
PA	+	+	+	+	+	+	+	+	+	-	+
Psy A	+	+	+	+	+	-	-	+	+	-	+
FA	+/-	+	+	-	-	-	-	-	+	-	+
N	+/-	+	+	-	+	-	-	-	+	+	+
SA	+	+	+	+	-	+	-	-	-	-	-
Setting	Hospital	ED and all clinical settings	ED	Clinical setting not specified	Health service setting not specified	Hospital	ED	ED	Dialysis dep.	Hospital	Clinical setting not specified
Duration (min)	120–180	12–15	< 1–3	10–15	unclear	unclear	unclear	unclear	unclear	unclear	unclear
Administered by	Healthcare professional	Nurse	ED nurse	Healthcare professional	Healthcare professional	Patient	unclear	ED nurse	Healthcare professional	Healthcare professional	Physician
Psychometrics	SE: 0.93 SPE: 0.98 Cronbα: 0.78–0.91	SE: 0.71 SPE: 0.93 Cronbα: 0.84	SE: 0.94 SPE: 0.90 Cronbα: missing	SE/SPE: missing Cronbα: 0.79–0.88	SE/SPE: missing Cronbα: missing	SE/SPE: missing Cronbα: missing	SE: 0.35 SPE: 0.71 Cronbα: missing	SE/SPE: missing Cronbα: missing	SE: 0.46 SPE: 0.73 Cronbα: missing	SE/SPE: overall value not mentioned. At cutoff point of 10 questions: SE: 0,11-0,20 SPE: 0,75–0,89 Cronbach α: missing	SE/SPE: missing Cronbα 0.73
Remarks	Long duration instrument, professional must be trained in use of instrument	Compre-hensive approach for screening suspected EA	Follow up by PhA if not confident on patient’s ability to report abuse; no questions only PhA on signs of SA	Limited in the EA context because not specifically developed for measuring this type of abuse	Assesses potential factors contributing to elder abuse	Directed at FeA and DV	Directed at DV	Directed at FeA and DV	Aimed at dialysis population	Directed at caregiver neglect	Component of a suggested protocol for the detection and assessment of EA

*Abbreviations*: + = type of elder abuse screened for; - = type of elder abuse not screened for; +/- = several instances of this type of elder abuse not identified; *EA *Elder abuse, *PA *Physical abuse, *Psy A *Psychological abuse, *FA *Financial abuse, *N *Neglect, *SA *Sexual abuse, *ED *Emergency department, *FeA *Female abuse, *DV *Domestic violence, *PVS *Partner violence screen, *PhA *Physical assement; 1 = Expanded Indicators of Abuse Screen; 2 = Elder Assessment Instrument; 3 = Emergency Department Senior Abuse Identification; 4 = Conflict Tactics Scale; 5 = Health, Attitudes Toward Aging, Living Arrangements, and Finances assessment; 6 = Index of spouse abuse-physical; 7 = Partner violence screen-physical; 8 = Two questions abuse screen; 9 = Questionnaire on elder abuse by family caregivers among older adults on hemodialysis; 10 = Clinical Signs of Neglect Scale; 11 = American medical association screen for various types of abuse and neglect; *SE *Sensitivity, *SPE *Specificity, *Cronbα *Cronbachs alpha

Although none of the eleven screening instruments and tools extracted from the literature review complied with all the predetermined screening tool selection criteria, the E-IOA, EAI and ED senior AID tool showed good psychometric properties and a reasonable feasibility. Unfortunately these instruments showed limitations with regard to a long duration of administration, the need for an extensive training to administer the instrument (E-IOA), the presence of many items (EAI), the need for a cognitive and/or physical assessment (the ED senior AID tool) or the need for the patient to be cognitively intact (EAI/E-IOA).

#### Field consultation

A total of 32 (44%) Dutch hospitals responded to the field consultation, consisting of large and small general teaching hospitals and one academic hospital (out of 7 academic hospitals present in the Netherlands). No one used a validated instrument for the detection of elder abuse. In general, the hospitals indicated the need for a simple, preferably single-question instrument, to screen multiple target groups for domestic violence and elder abuse.

#### Early warning tool ERASE

The development group formulated a single awareness question as a starting question that encompassed all forms of elder abuse. This question was neutrally formulated and without any reference to age: “Are you concerned about neglect or abuse?” Subsequently, six signalling questions were developed to collect information on observed signs and symptoms of elder abuse and neglect (see Additional file 3). These consisted of questions on the interaction with the informal caregiver, signs of overburdening and derailment of informal care, signs of neglect and unexplained delay in seeking medical attention, signs or suspicion of inflicted injury and finally, other signs with regard to financial, psychological and sexual abuse.

The ERASE tool was administered as a prescreener, during the beginning of the first clinical encounter. The respondents filled out the ERASE tool together with other instruments in the electronic medical record (EMR) such as a pain score, a delirium score etc. The ERASE tool was incorporated in the EMR, and applied in patients aged 70 years and older. If the answer on the starting question yielded a “yes”, this was considered as a positive screening on the ERASE tool. In case of a positive screening, furthermore the signalling questions of the ERASE tool described which potential signals were detected. The signalling questions with “ pop-up” examples of elder abuse were always visible when the ERASE starting question was answered with a yes.

### Acceptability and appropriateness outcomes of the ERASE tool

A total of *n* = 28 respondents participated in the feasibility study. The respondents assessed 386 older patients using the ERASE tool. which yielded a positive score on elder abuse in 15 patients (3,9%; 95% Confidence interval 2.4–6.3%). Completing the ERASE tool took an average of 7.15 (*SD* = *6.77*) minutes. Twenty-five respondents completed the evaluation questionnaire, three respondents were unable to do so due to sick leave, prolonged illness and holidays, respectively. The respondents had different professional functions. The largest group professionals (*n* = 13) consisted of nurses of the ED, followed by physicians (including emergency physicians and clinical geriatricians (*n* = 7)), and then nurse specialists of the Geriatric Department (*n* = 4). Female respondents were in the majority (*n* = 20). One person did not notate his/her function and gender. Most of the respondents (*n* = 23/25; 92%) (totally) agreed that the ERASE starting question was formulated in an understandable way. They mentioned that the signalling questions were clear and were accompanied by some helpful examples of elder abuse to stimulate the thinking process of respondents and prevent anyone from overlooking a certain category of elder abuse. In total, 88% of the respondents (totally) agreed that the ERASE starting question did increase awareness on the topic of elder abuse, and 88% also (totally) agreed that the ERASE starting question and signalling questions helped to clearly and systematically identify and map out signals of elder abuse. For more details regarding the answers on the questionnaire see Additional file 4.

### Modification to the early warning tool ERASE

Some respondents did mention that the given options (yes and no) for answering the starting question were (too) strict, which made it difficult to provide a positive score, as on admission a suspicion of elder abuse cannot be ruled. Therefore the ERASE starting question was modified by adding the option 'doubt' to the answer options 'yes' and 'no'. Both the “yes” or “doubt” responses to the starting question were considered as a positive screening on the ERASE tool. No further modifications were done. See Additional file 5 for further clarification on the processing of comments. See Table 2 for the final early warning tool ERASE.

**Table 2:** Early warning tool ERASE final version

	Item	
ERASE starting question		Are you concerned about neglect or abuse?
		□ No □ Yes □ Doubt
ERASE signalling questions:		
	SQ1	Is the response and interaction between the elder and the caregiver/family appropriate?
		□ No □ Yes □ Clarification:
	SQ2	Are there signs of overburdening and derailment of informal care?
		□ No □ Yes □ Clarification:
		Clickable pop-up: possible signs: frustration, compassion fatigue, transgressive behavior toward elder or caregiver
	SQ3	Is there an unexplained delay in seeking medical attention?
		□ No □ Yes □ Clarification:
	SQ4	Is there a suspicion of inflicted injury?
		□ No □ Yes □ Clarification:
		Clickable pop-up: possible signs: unexplained bruising, injury of different date, inflicted injury does not fit to given history
	SQ5	Are there any signs of neglect?
		□ No □ Yes □ Clarification:
		Clickable pop-up: possible signs: malnutrition, untreated pressure sores, unkempt wounds, poor physical hygiene
	SQ6	Are there any other signs?
		□ No □ Yes □ Clarification
		Clickable pop-up: possible signs: abuse of PGB^a^, lack of standard (medical) facilities, anxiety, depressive symptoms, behavioral changes, unexplained bruising in genital area, unexplained sexual transmitted diseases

^a^PGB is a personal budget under the Social Support Act for support from the municipal authorities

## Discussion

In this study, we have described the development process and feasibility outcomes of ERASE, an early warning tool for the detection of elder abuse in the ED. ERASE is administered by the healthcare professional in persons 70 years and older upon admission to the ED, and is guided by an initial gut feeling question and six signalling questions. The pilot study showed that the ERASE tool raised the recognition of healthcare professionals for elder abuse, and the tool was evaluated as acceptable and appropriate to use in the ED setting. The number of six signalling questions seemed to be a good balance between comprehensiveness and the desired brevity of the tool. The respondents requested to add the answer option doubt to the gut feeling question, in order to further clear up and investigate potential elder abuse. Doubt is seen as an answer that indicates that the healthcare professional is not sure of the presence of elder abuse. When comparing ERASE to other tools in the literature that involve the input of the gut feeling of the healthcare professional and try to flag all major forms of elder abuse, the EASI and BASE tools [13] could be comparable to ERASE, although both tools were not specifically developed and validated for the ED setting. The EASI tool is developed for the family medicine setting, and is aimed to be administered by physicians to cognitively intact seniors, while ERASE focuses on all healthcare professionals in the ED (e.g. nurses, residents and physicians) and also addresses seniors with cognitive problems. The BASE tool is developed to be administered by healthcare professionals after (extensive) training, focuses on the caregiver and omits sexual abuse as a form of elder abuse. The ERASE tool takes only a few minutes to administer, the focus is directed solely on the patient (because the caregiver is not always present at the ED), it covers all forms of elder abuse and no extensive training is required.

Three other interesting tools to identify elder abuse in the ED/hospital setting have been developed since our literature review (until May 2019). This encompasses the EM-SART tool [27], the REAGERA-S instrument [28] and the VOICES tool [29]. In comparison to the ERASE tool, the REAGERA-S instrument and the VOICES tool have to be self-administered by the patient. Frail elderly at risk for elder abuse, especially during admission in an acute emergency care setting, often have insufficient physical and/or cognitive capacity to self-administer the questions of the instrument. Therefore, the ERASE tool was developed to be administered by healthcare professional themselves. The EM-SART tool, like the ERASE tool, encompasses a pre-screen section to appeal on concerns for abuse. However, the pre-screen section of EM-SART tool exists of multiple screening sections, including a cognitive screening which makes the instrument very lengthy in use. Furthermore, specific training is necessary to administer the tool.

The use of the ERASE early warning tool offers a simple opportunity to support healthcare professionals to identify which patients, from all the older patients that are admitted to the ED on a daily basis, require more detailed questioning with regard to a suspicion of elder abuse. However, applying a tool to increase awareness on signs of elder abuse is not enough. Healthcare professionals need to know what to do when potential elder abuse is ascertained [30]. In the Netherlands a healthcare professional is obliged to act according to the The Domestic Violence and Child Abuse Reporting Code. A reporting code with five steps has to be followed to decide whether it is necessary to report to Adult Protective Services (APS). Furthermore, it also remains important to discuss (possible) cases of elder abuse in a multidisciplinary setting. Having some sense of elder abuse cases can help physicians to learn from the kind of cases that might present themselves at an ED [27]. The implementation of screening on elder abuse thus not only includes an early warning tool, but also necessitates the creation of a guide on how to act if abuse is suspected [30].

## Conclusion

Our study showed that the ERASE is an acceptable and appropriate early warning tool, an initial gut feeling question followed by six signalling questions, to support the recognition of elder abuse in healthcare professionals in the ED setting. The next step will be to investigate the reliability and validity of the ERASE tool.

### Strengths and Limitations

The strength of the methodological development of the ERASE tool was that it was based on a systematic literature review combined with field consultation and expert-based opinion. We incorporated the field consultation adjacent to the literature review in order to link up the experiences and needs of 32 Dutch hospitals. Furthermore, the tool was directed at a clinician’s internal sense of alarm or gut feeling, which has been studied as an important diagnostic compass in physicians and nurses [31, 32].

Limitations of this study were that the systematic literature review was not reported according to PROSPERO criteria, although this was justifiable because it was part of the methodology of this development and feasibility study. Moreover, the patients’ perspective was not included in the development of ERASE tool and the pilot study because we were unable to find victims of elder abuse who were willing to participate in this study. The COVID-19 pandemic could have had an impact on the recruitment of patients in the ED, although we do not think that this has affected the outcome of this study because it was a small pilot focused on feasibility outcomes. Although, on the one hand, older persons were more at risk for elder abuse during the pandemic, on the other hand, fewer older persons visited the ED during this period. Furthermore, the ERASE tool was implemented during all shifts (24/7 h a week), we do not have information on any selection bias of use during shifts.

A final limitation could be that early adapters on the awareness of elder abuse are possibly over-represented in the convenience sample. Respondents in the limited sample may have already been interested in the elder abuse topic, leading to their willingness to participate and this could have induced bias in our results. However, we asked the local officers on domestic violence and elder abuse to also approach critical respondents who are not comfortable with the increasing use of instruments and tools to screen on vulnerabilities in older persons (such as the instruments for pain, cognitive- and nutritional status etc.). Baseline information on the knowledge and attitude on the recognition of elder abuse from the respondents was not collected.

### Supplementary Information

**Additional file 1. **Search strategy databases MEDLINE, Embase and CINAHL.

**Additional file 2. **Questionnaire respondents ERASE tool.

**Additional file 3. **ERASE tool draft version.

**Additional file 4. **Multiple choice & Free text answers questionnaire ERASE tool.

**Additional file 5. **ERASE tool draft version including comments.

## Data Availability

The data that support the findings of this study are available from the corresponding author, Miriam E. van Houten, upon request.
