# Supplementary material for: ERASE: a feasible early warning tool for elder abuse, developed for use in the Dutch emergency department
Source: BMC Emerg Med. 2024 Apr 3;24:52. doi: 10.1186/s12873-024-00971-6 (PMC10988976; doi:10.1186/s12873-024-00971-6)
Supplement: Supplementary file 1 — Additional file 1. Search strategy databases MEDLINE, Embase and CINAHL. [file 12873_2024_971_MOESM1_ESM.docx]

**Additional file 1. Search strategy databases**

**Medline 2005-13 mei 2019**

1 elder abuse/ or physical abuse/ or violence/ or domestic violence/ or torture/ or Patient Isolation/ or Restraint, Physical/ or fraud/ or homicide/ or sex offenses/ or rape/ or theft/ or Contusions/

2 ((mental* or physical or verbal or emotional or financial or sexual or psychological or material or elder*) adj2 (harm or abus*)).ti,ab,kf.

3 (neglect* or ill-treat* or maltreat* or mistreat* or fraud* or assault* or crime* or violen* or bully* or intimidat* or aggression or coerc* or extort* or stigmati* or ostraci* or bruis*).ti,ab,kf.

4 1 or 2 or 3

5 exp *Aged/ or exp *geriatrics/ or exp Geriatric Assessment/ or exp Home Nursing/ or exp *Alzheimer Disease/ or exp Homes for the Aged/ or exp Nursing Homes/ (184866)

6 (community-dwelling or alzheimer* or frail* or ageing or aging or dement* or psychogeriatric* or geriatric* or elder* or "cognitive impairment*" or old-age* or pensioner* or retire*).ti,ab,kf. or (aged or old or older).ti. (875513)

7 5 or 6

8 elder abuse/

9 (4 and 7) or 8

10 limit 9 to (dutch or english)

11 exp "surveys and questionnaires"/ or "Mass Screening"/ or Elder Abuse/di, pc or Risk Assessment/ or risk factors/ or exp Geriatric Assessment/

12 (red adj flag*).ti,ab.

13 (Screen* or (Risk* adj2 Assess*) or checklist* or tool* or questionnaire* or detect* or identif* or predict*).ti,ab,kf.

14 ((suspic* or sign* or symptom*) adj3 (abuse or mistreatment or bruis*)).ti,ab,kf.

15 Physical Examination/

16 head-to-toe.ti,ab,kf.

17 Examination.ti,ab,kf.

18 forensic medicine/ or forensic pathology/

19 (forensic* or legal*).ti,ab,kf.

20 or/11-19

21 10 and 20

22 limit 21 to yr="2005 -13 may 2019"

23 remove duplicates from 22

24 (meta-analysis/ or meta-analysis as topic/ or (meta adj analy$).tw. or ((systematic* or literature) adj2 review$1).tw. or (systematic adj overview$1).tw. or exp "Review Literature as Topic"/ or cochrane.ab. or cochrane.jw. or embase.ab. or medline.ab. or (psychlit or psyclit).ab. or (cinahl or cinhal).ab. or cancerlit.ab. or ((selection criteria or data extraction).ab. and "review"/)) not (Comment/ or Editorial/ or Letter/ or (animals/ not humans/))

25 23 and 24

26 23 not 24

**Embase 2005-13 mei 2019**

1 'elder abuse'/exp/mj OR ('domestic violence'/exp/mj OR 'torture'/exp/mj OR 'patient isolation'/exp/mj OR 'sexual assault'/exp/mj OR 'sexual coercion'/exp/mj OR 'theft'/exp/mj OR 'contusion'/exp/mj OR ((mental* OR physical OR verbal OR emotional OR financial OR sexual OR psychological OR material OR elder*) NEAR/2 (harm OR abus*)):ti,ab OR neglect*:ti,ab OR 'ill treat*':ti,ab OR maltreat*:ti,ab OR mistreat*:ti,ab OR fraud*:ti,ab OR assault*:ti,ab OR crime*:ti,ab OR violen*:ti,ab OR bully*:ti,ab OR intimidat*:ti,ab OR aggression:ti,ab OR coerc*:ti,ab OR extort*:ti,ab OR stigmati*:ti,ab OR ostraci*:ti,ab OR bruis*:ti,ab

2 (elderly:ab,ti OR 'community dwelling':ab,ti OR geriatric:ab,ti OR 'mini mental state':ab,ti OR alzheimer:ab,ti OR alzheimers:ab,ti OR mmse:ab,ti OR caregivers:ab,ti OR falls:ab,ti OR adl:ab,ti OR frailty:ab,ti OR gds:ab,ti OR ageing:ab,ti OR elders:ab,ti OR frail:ab,ti OR mci:ab,ti OR demented:ab,ti OR psychogeriatrics:ab,ti OR 'cognitive impairment':ab,ti OR 'postmenopausal women':ab,ti OR comorbidities:ab,ti OR 'geriatric assessment'/exp OR 'nursing home'/exp OR 'frail elderly'/exp OR 'alzheimer disease'/exp OR 'cognitive defect'/exp OR 'home for the aged'/exp OR 'aged'/exp/mj)) OR (elder NEAR/3 (abus* OR neglect*)):ti,ab

3 ([dutch]/lim OR [english]/lim) AND [embase]/lim

4 ('questionnaire'/exp/mj OR 'mass screening'/exp/mj OR 'risk factor'/exp/mj OR 'risk assessment'/exp/mj OR 'geriatric assessment'/exp/mj OR 'physical examination'/exp/mj OR (red NEAR/1 flag*):ti,ab OR screen*:ti,ab OR (risk* NEAR/2 assess*):ti,ab OR checklist*:ti,ab OR tool*:ti,ab OR questionnaire*:ti,ab OR detect*:ti,ab OR identif*:ti,ab OR predict*:ti,ab OR ((suspic* OR sign* OR symptom*) NEAR/3 (abuse OR mistreatment OR bruis*)):ti,ab OR 'head to toe':ti,ab OR examination:ti,ab OR forensic*:ti,ab OR legal*:ti,ab OR 'forensic medicine'/exp/mj) NOT 'conference abstract':it

5 [2005-2019]/py

6 'meta analysis'/de OR cochrane:ab OR embase:ab OR psycinfo:ab OR cinahl:ab OR medline:ab OR (systematic NEAR/1 (review OR overview)):ab,ti OR (meta NEAR/1 analy*):ab,ti OR metaanalys*:ab,ti OR 'data extraction':ab OR cochrane:jt OR 'systematic review'/de NOT ('animal experiment'/exp OR 'animal model'/exp OR 'nonhuman'/exp NOT

7 1 AND 2 AND 3 AND 4 AND 5 AND 6

**CINAHL 2005-13 mei 2019**

S1 (MH "Elder Abuse")

S2 TI ( elder N3 (abus* or neglect or maltreatment or illtreatment)) ) OR AB ( elder N3 (abus* or neglect or maltreatment or illtreatment)) )

S3 S1 or S2

S4 (MH "Health Screening") OR (MH "Geriatric Assessment")

S5 (MH "Questionnaires+")

S6 (MH "Physical Examination+")

S7 (MH "Forensic Medicine+")

S8 TI ( (Screen* or (Risk* N2 Assess*) or checklist* or tool* or questionnaire* or detect* or identif* or predict*) ) OR AB ( (Screen* or (Risk* N2 Assess*) or checklist* or tool* or questionnaire* or detect* or identif* or predict*) )

S9 TI ( (forensic or legal or examination or (red n1 flag*)) ) OR AB ( (forensic or legal or examination or (red n1 flag*)) )

S10 S4 OR S5 OR S6 OR S7 OR S8 OR S9

S11 S3 AND S10

S12 (MH "Meta Analysis") or TX (meta-analy* or metanaly* or metaanaly* or meta analy*) or TX (systematic* N5 review*) or (evidence* N5 review*) or (methodol* N5 review*) or (quantitativ* N5 review*) or TX (systematic* N5 overview*) or (evidence* N5 overview*) or (methodol* N5 overview*) or (quantitativ* N5 overview*) or TX (systematic* N5 survey*) or (evidence* N5 survey*) or (methodol* N5 survey*) or (quantitativ* N5 survey*) or TX (systematic* N5 overview*) or (evidence* N5 overview*) or (methodol* N5 overview*) OR (quantitativ* N5 overview*) OR TX (pool* N2 data) OR (combined N2 data) OR (combining N2 data) OR (pool* N2 trials) OR (combined N2 trials) OR (combining N2 trials) OR (pool* N2 studies) OR (combined N2 studies) OR (combining N2 studies) OR (pool* N2 results) OR (combined N2 results) OR (combining N2 results)

S13 S11 AND S12

S14 S11 NOT S12 cannot be performed because missing “NOT” boolean operator in this version of CINAHL
