# Supplementary material for: ERASE: a feasible early warning tool for elder abuse, developed for use in the Dutch emergency department
Source: BMC Emerg Med. 2024 Apr 3;24:52. doi: 10.1186/s12873-024-00971-6 (PMC10988976; doi:10.1186/s12873-024-00971-6)
Supplement: Supplementary file 2 — Additional file 2. Questionnaire respondents ERASE tool. [file 12873_2024_971_MOESM2_ESM.docx]

**Additional file 2. Questionnaire respondents ERASE tool**

**General questions:**

**My profession is:**

| Answer |
| --- |
| ED nurse |
| Geriatric nurse |
| Geriatric nurse specialist |
| ED-physician |
| Clinical geriatrician |
| General physicians |

**I am working in the following hospital:**

| Answer |
| --- |
| H1 |
| H2 |
| H3 |

**My gender is:**

| Answer |
| --- |
| Male |
| Female |

**Q1: The starting question has made me more aware of the issue of elder abuse.**

| Answer |
| --- |
| I totally agree |
| Agree |
| Neither agree nor disagree |
| Disagree |
| I totally disagree |

**Q2: The starting question is worded in an understandable way for me.**

| Answer |
| --- |
| I totally agree |
| Agree |
| Neither agree nor disagree |
| Disagree |
| I totally disagree |

**Q3: The six signalling questions help me to identify the signals clearly and systematically**

| Answer |
| --- |
| I totally agree |
| Agree |
| Neither agree nor disagree |
| Disagree |
| I totally disagree |

**Q4. I find the question "is the response and interaction between the elder and the caregiver/family appropriate" relevant in recognizing elder abuse.**

| Answer |
| --- |
| Yes, please explain further |
| No |

**Q5. I find the question "Are there signs of overload and derailment of informal care?" relevant in recognizing elder abuse.**

| Answer |
| --- |
| Yes, please explain further |
| No |

**Q6: I find the question " is there an unexplained delay in seeking medical attention" relevant in recognizing elder abuse.**

| Answer |
| --- |
| Yes, please explain further |
| No |

**Q7: I find the question " is there a suspicion of inflicted injury" relevant in recognizing elder abuse.**

| Answer |
| --- |
| Yes, please explain further |
| No |

**Q8: I find the question "are there signs of neglect" relevant in recognizing elder abuse.**

| Answer |
| --- |
| Yes, please explain further |
| No |

**Q9. I find the question "are there other signs" relevant in recognizing elder abuse.**

| Answer |
| --- |
| Yes, please explain further |
| No |

**Q10: Do you miss any signalling questions?**

| Answer |
| --- |
| Yes, please explain further |
| No |

**Q11: The starting question with the six signalling questions help me to systematically identify elder abuse.**

| Answer |
| --- |
| I totally agree |
| Agree |
| Neither agree nor disagree |
| Disagree |
| I totally disagree |

**Q12: How much time does it take to complete the ERASE tool?**

**Q13: The ERASE tool is easy to find in the EMR.**

| Answer |
| --- |
| I totally agree |
| Agree |
| Neither agree nor disagree |
| Disagree |
| I totally disagree |
