# Supplementary material for: ERASE: a feasible early warning tool for elder abuse, developed for use in the Dutch emergency department
Source: BMC Emerg Med. 2024 Apr 3;24:52. doi: 10.1186/s12873-024-00971-6 (PMC10988976; doi:10.1186/s12873-024-00971-6)
Supplement: Supplementary file 3 — Additional file 3. ERASE tool draft version. [file 12873_2024_971_MOESM3_ESM.docx]

**Additional file 3. ERASE tool draft version**

| **Area of abuse** | **Item** | **Version 1** |
| --- | --- | --- |
| **ERASE starting question** |  | Are you concerned about neglect or abuse? Answer options: □ Yes □ No |
| **ERASE signalling questions** |  |  |
|  | SQ1 | Is the response and interaction between the elder and the caregiver/family appropriate?  □ No □ Yes Clarification: |
|  | SQ2 | Are there signs of overburdening and derailment of informal care?  □ No □ Yes Clarification:  Clickable pop-up: possible signs: frustration, compassion fatigue, transgressive behavior toward elder or caregiver |
|  | SQ3 | Is there an unexplained delay in seeking medical attention?  □ No □ Yes Clarification: |
|  | SQ4 | Is there a suspicion of inflicted injury?  □ No □ Yes Clarification::  Clickable pop-up: possible signs: unexplained bruising, injury of different date, inflicted injury does not fit to given history |
|  | SQ5 | Are there any signs of neglect?  □ No □ Yes Clarification:  Clickable pop-up: possible signs: malnutrition, untreated pressure sores, unkempt wounds, poor physical hygiene |
|  | SQ6 | Are there any other signs?  □ No □ Yes Clarification:  Clickable pop-up: possible signs: abuse of PGB*, lack of standard (medical) facilities, anxiety, depressive symptoms, behavioral changes, unexplained bruising in genital area, unexplained sexual transmitted diseases |

* PGB is a personal budget under the Social Support Act for support from the municipal authorities
