# Supplementary material for: ERASE: a feasible early warning tool for elder abuse, developed for use in the Dutch emergency department
Source: BMC Emerg Med. 2024 Apr 3;24:52. doi: 10.1186/s12873-024-00971-6 (PMC10988976; doi:10.1186/s12873-024-00971-6)
Supplement: Supplementary file 4 — Additional file 4. Multiple choice & Free text answers questionnaire ERASE tool. [file 12873_2024_971_MOESM4_ESM.docx]

**Additional file 4 Multiple choice and Free text answers questionnaire ERASE**

**General questions:**

**My profession is:**

| Answer | Number | Percentage |
| --- | --- | --- |
| ED nurse | **13** | **54,17** |
| Geriatric nurse | **2** | **8,33** |
| Geriatric nurse specialist | **2** | **8,33** |
| ED-physician | **1** | **4,17** |
| Clinical geriatrician | **2** | **8,33** |
| General physicians | **4** | **16,67** |
|  | **24** |  |

**I am working in the following hospital:**

| Answer | Number | Percentage |
| --- | --- | --- |
| H1 | 11 | **44** |
| H2 | **7** | **28** |
| H3 | **7** | **28** |
|  | **25** |  |

**My gender is:**

| Answer | Number | Percentage |
| --- | --- | --- |
| Male | 4 | 16,67 |
| Female | 20 | 83,33 |
|  | **24** |  |

**Q1: The signalling question has made me more aware of the issue of elder abuse.**

| Answer | Number | Percentage |
| --- | --- | --- |
| I totally agree | **11** | **44** |
| Agree | **11** | **44** |
| Neither agree nor disagree | **3** | **12** |
| Disagree | **0** | **0** |
| I totally disagree | **0** | **0** |
|  | **25** |  |

| I totally agree | Question is totally clear |
| --- | --- |
| I totally agree | The difference between neglect with cognitive problems and without cognitive problems and the role of the professional in this |
| Neither agree nor disagree | We are trained to recognize signs of elder abuse. As a result, you are always (unconsciously) busy with screening. |
| Agree | Handy that you can also open the fact sheet elder abuse, so that you know what to think about. You are alerted. |
| Neither agree nor disagree | In the ED I am always alert for possible signs of abuse/neglect. |
| Neither agree nor disagree | Sometimes you notice that something is not right, often the injury is accidental |
| I totally agree | More consciously and in my opinion, this question also makes it more accessible to do something with your suspicion. |
| Agree | I was already consciously seen as a member of the domestic violence and elder abuse committee |
| I totally agree | I pay better attention now |
| Agree | I think it's nonsense that we have to register so much, it just takes time and it doesn't yield us anything. I'm not in favor of all that fuss! |

**Q2: The signalling question is worded in an understandable way for me.**

| Answer | Number | Percentage |
| --- | --- | --- |
| I totally agree | **12** | **48** |
| Agree | **11** | **44** |
| Neither agree nor disagree | **0** | **0** |
| Disagree | **2** | **8** |
| I totally disagree | **0** | **0** |
|  | **25** |  |

| Agree | Perhaps a brief explanation could be added. |
| --- | --- |
| Agree | Clear, concrete and short. very nice. |
| Agree | There are situations in which there are no concerns because the patient is already embedded in care, where there is already attention (and guidance) for the situation |
| Agree | The questions are quite definite, which makes it sometimes difficult to answer positively because it is often based on a suspicion |
| I totally agree | Clear question and with regard to factsheet you can look at the factsheet to refresh your memory |
| Agree | Perhaps (self) neglect is even more obvious to add to the signallling question |

**Q3: The six signalling questions help me to identify the signals clearly and systematically**

| Answer | Number | Percentage |
| --- | --- | --- |
| I totally agree | **9** | **36** |
| Agree | **13** | **52** |
| Neither agree nor disagree | **3** | **12** |
| Disagree | **0** | **0** |
| I totally disagree | **0** | **0** |
|  | **25** |  |

| I totally agree | Clear questions |
| --- | --- |
| Neither agree nor disagree | Have not used these |
| I totally agree | Clear questions with some examples, so that you are well aware of what to think about. |
| I totally agree | This will make you not forget a category. |
| Agree | What is meant with the signalling question: are there other signals? |

**Q4. I find the question "is the response and interaction between the elder and the caregiver/family appropriate" relevant in recognizing elder abuse.**

| Answer | Number | Percentage |
| --- | --- | --- |
| Yes, please explain further | **23** | **88** |
| No | **2** | **12** |
|  | **25** |  |

| Yes | Just like with children, this gives clues about the relationship between the two and what the atmosphere is like between them. In case of 'inappropriate' interaction, there is a higher suspicion of elder abuse |
| --- | --- |
| Yes | A deviant interaction may indicate a disturbed relationship between the two |
| Yes | As a nurse, you can already learn a lot from the interaction so that you can ask further questions where necessary |
| Yes | This makes you alert to how communication is going and the interaction between family / informal care. |
| Yes | I think the question is relevant, but the question is subjective. The care provider therefore indicates whether he/she finds the interaction appropriate and it therefore depends on his or her own frameworks. |
| Yes | Can be relevant if there are specific, inappropriate (re)actions, but you can also get bogged down due to the large number of questions |
| Yes | Difficult because it is of course subjective and it is quite a big judgment |
| Yes | The response and interaction gives me a good picture of the situation and tools to make a report or not. |
| Yes | It often says something about the dynamics, and that there is a lot more to it than meets the eye |
| Yes | You can see the interaction between often carers / family members, so that you can always get a story there about how the home situation is |
| Yes | But problem is that sometimes you understand it so well and then whitewash it. |
| Yes | Always good to think about the interaction. |
| Yes | Treatment and mutual communication give many important signals |
| Yes | Sometimes the reaction is appropriate for the interaction, but it is also an undesirable reaction. so I don't think it's the right wording. |
| Yes | Think that is certainly relevant, however, in the case of structural abuse (deliberate, e.g. financial exploitation) care must be taken that a "normal" interaction is acted on deliberately by the perpetrator. |
| Yes | Often a sign of overload between caregiver and patient |
| Yes | Observation is important |
| Yes | The question is correctly formulated |

**Q5. I find the question "Are there signs of overload and derailment of informal care?" relevant in recognizing elder abuse.**

| Answer | Number | Percentage |
| --- | --- | --- |
| Yes, please explain further | **23** | **92** |
| No | **2** | **8** |
|  | **25** |  |

| Yes | If there are indications of overload or derailment, I can imagine that the risk of elder abuse is higher. On the other hand, this is also a sign of commitment. |
| --- | --- |
| Yes | Overburdened informal caregivers can derail their stress coping, which can lead to abuse and/or neglect |
| Yes | I find that there is often a visible connection between neglect and overburdened informal carers. |
| Yes | You are made more alert to this because of this question |
| Yes | Yes, I think the question is relevant. This is a common signal in elder abuse. |
| Yes | Overload of informal care can indicate that care can no longer be provided properly, then a care shortage may arise |
| Yes | A common problem that you should always be aware of. It's good that this is a specific question. |
| Yes | Yes, very important, whether there is an overburdened caregiver |
| Yes | This may be a first sign of the poorer functioning of the elderly. |
| Yes | This is also a form of elder abuse. |
| Yes | The carer often likes that we raise the matter and to be heard |
| Yes | Important to recognize it, taking into account all the factors that play a role. |
| Yes | Makes it easier to start a conversation about your concerns |
| Yes | Clarifies personal circumstances, good to be empathetic but it is still abuse |
| Yes | The question is correctly formulated |

**Free text Q6: I find the question " is there an unexplained delay in seeking medical attention" relevant in recognizing elder abuse.**

| Answer | Number | Percentage |
| --- | --- | --- |
| Yes, please explain further | **22** | **91.67** |
| No | **2** | **8.33** |
|  | **24** |  |

| Yes | In the event of a delay, you may wonder whether this is because the patient trivializes the complaints or whether the informal caregiver did not want to cooperate |
| --- | --- |
| Yes | An unexplained delay can erase traces of abuse and or indicate neglect |
| Yes | When informal carers or others are aware, but do not take action, this falls under neglect/abuse |
| Yes | If someone has had a fracture for days and comes to the hospital only days later, that is a reason to check carefully why this is (and this also applies to other complaints of course) |
| Yes | You have to ask yourself why the informal carer did not call for help earlier, what is the underlying cause of this |
| Yes | Although this is again subjective, because people themselves sometimes do not want to see a doctor and this is therefore a conscious choice |
| Yes | Good to find out why there is a delay, so that you know what the patient's caregiving looks like |
| Yes | A delay could indicate elder abuse. |
| Yes | Severe neglect |
| Yes | Moderately relevant. delay can often be explained in different ways. but good to be aware of it. |
| Yes | Relevant, but in some cases difficult to estimate |
| Yes | If an informal caregiver is a "knowing" perpetrator of elder abuse, there may be a delay in trying to mask recognition of abuse |
| Yes | The question is correctly formulated |

**Q7: I find the question " is there a suspicion of inflicted injury" relevant in recognizing elder abuse.**

| Answer | Number | Percentage |
| --- | --- | --- |
| Yes, please explain further | **24** | **96** |
| No | **1** | **4** |
|  | **25** |  |

| Yes | Injury is indicative of abuse |
| --- | --- |
| Yes | Unexplained hematomas in places where people are grabbed may indicate abuse |
| Yes | It goes without saying that all questions can help to clarify the situation and to deploy adequate care/actions |
| Yes | Good to fully examine the patient and write down findings. |
| Yes | Injuries that don't match the problem |
| Yes | Yes and no, if you need one more question to remember if you suspect injury.... |
| Yes | Ditto, one of the possible forms of elder abuse |
| Yes | Injury is sometimes difficult to recognize as abuse in the elderly |
| Yes | The question is correctly formulated |

**Q8: I find the question "are there signs of neglect" relevant in recognizing elder abuse.**

| Answer | Number | Percentage |
| --- | --- | --- |
| Yes, please explain further | **22** | **88** |
| No | **3** | **12** |
|  | **25** |  |

| Yes | Especially in patients who are ADL dependent |
| --- | --- |
| Yes | In case of neglect, there are people in the environment of the elderly who have not taken on responsibilities such as arranging and/or carrying out physical care |
| No | Perhaps too broad a concept. |
| Yes | Neglect can indicate that the caregiver is overloaded |
| Yes | Related to elder abuse |
| Yes | Good to check what you think are signs of neglect. |
| Yes | Delayed injury that has not been acted upon |
| Yes | Often neglect is the first or only signal. |
| Yes | Certainly important, the question contains clear possible signals |
| Yes | Consider whether the patient is self-neglecting or whether others may be involved |
| Yes | The question is correctly formulated |

**Q9. I find the question "are there other signs" relevant in recognizing elder abuse.**

| Answer | Number | Percentage |
| --- | --- | --- |
| Yes, please explain further | **17** | **68** |
| No | **8** | **32** |
|  | **25** |  |

| Yes | This gives room for your own insight and makes you think about it better |
| --- | --- |
| Yes | There may be examples for this question. |
| No | In order not to make the list endless, I advocate clustering some questions so that a workable screening list is created that is not endless |
| No | Difficult, because then you don't really know what is meant. and therefore do not know what to signal |
| Yes | It does help that there are examples |
| Yes | You can place the other answers here. |
| Yes | Sometimes a ′′ gut′′ feeling can't be captured in other questions |
| No | I don't quite understand what this means, example? Is this about, for example, stealing money, etc.? useful to write example |

**Q10: Do you miss any signalling questions?**

| Answer | Number | Percentage |
| --- | --- | --- |
| Yes, please explain further | **1** | **4** |
| No | **24** | **96** |
|  | **25** |  |

| No | Perhaps whether there is adequate help at home and if not, why. Although this may not be directly assessable in the ED and more something for chronic care. I think the questions are complete. |
| --- | --- |
| No | It could also be more specific to ask are there signals present and then check yes/no, if yes, check which signals are present. |

**Q11: The signaling tool with the six signalling questions helps me to systematically identify elder abuse.**

| Answer | Number | Percentage |
| --- | --- | --- |
| I totally agree | **11** | **44** |
| Agree | **11** | **44** |
| Neither agree nor disagree | **3** | **12** |
| Disagree | **0** | **0** |
| I totally disagree | **0** | **0** |
|  | **25** |  |

| I totally agree | Clear signaling instrument with clear questions. Very compact, works fine. |
| --- | --- |
| Neither agree nor disagree | I find the six signalling questions well applicable |
| Agree | See above |
| Neither agree nor disagree | It is very extensive to complete for each included patient. Especially if there are no clues. I think one question is enough. |
| I totally agree | Clear and organized |

**Q12: How much time does it take to complete the signalling tool and possibly the signalling questions?**

| About a minute, I don't think it's too long anyway |
| --- |
| At least 10 minutes |
| 5 minutes |
| Maximum 5 minutes. |
| 1 minute |
| At most screenings I was done quickly, less than 1 minute because the first answer was already 'no'. |
| 30 minutes |
| Depends if you have any suspicions. It's nice that for patients where there is no suspicion, only one question has to be filled in. If you do have suspicions, it takes about 10 to 15 minutes |
| 5-10 minutes |
| A lot, you can answer many questions extensively and come up with a lot, I myself tend to write out the whole problem with the first question |
| About 7 minutes, which I think is a lot. This is because you have to think a lot about the hard questions |
| Some minutes |
| Maximum 5 minutes |
| If no signals are present within minute. At full around 4 minutes. It will probably also go faster if you use it more. There are still many questions to go through. |
| 3 minutes |
| 15 minutes |
| 3 tot 5 minutes |
| Depending on the case, but quite a long time! it's always disheartening to think about. |
| 5 minutes |
| I have completed the vignette cases and cannot estimate how much time this would take if it had been a "live" patient |
| I think 3 minutes, but if I have to call the APS and speak to the official then much longer, depending on how easily accessible they are |
| 15 minutes |
| 5 minutes |
| 10 minutes |
| 5 minutes |

**Q13: The signalling tool is easy to find in the EMR.**

| Answer | Number | Percentage |
| --- | --- | --- |
| I totally agree | **11** | **44** |
| Agree | **10** | **40** |
| Neither agree nor disagree | **3** | **12** |
| Disagree | **1** | **4** |
| I totally disagree | **0** | **0** |
|  | **25** |  |

| I totally agree | it ''pops up'' when someone has a certain age, I do hear from others that this happens to some and not to others |
| --- | --- |
| I totally agree | If you know where to find it. |
| I totally agree | Clearly and in the correct order of discussion. |
| I totally agree | clearly visible as it is built into our EMR in the test environment |
