# Supplementary material for: ERASE: a feasible early warning tool for elder abuse, developed for use in the Dutch emergency department
Source: BMC Emerg Med. 2024 Apr 3;24:52. doi: 10.1186/s12873-024-00971-6 (PMC10988976; doi:10.1186/s12873-024-00971-6)
Supplement: Supplementary file 5 — Additional file 5. ERASE tool draft version including comments. [file 12873_2024_971_MOESM5_ESM.docx]

Additional file 5. ERASE tool draft version including comments

| **Area of abuse** | **Item** | **Modifications from version 1** | **Comments** |
| --- | --- | --- | --- |
| **ERASE starting question** |  | Added the option 'doubt' to the answer options 'yes' and 'no' | It was mentioned by one respondent that the answer options yes and no of the ERASE starting question were (too) strict, which made it more difficult to score positively. Although the comment was only given by 2 respondents, the project group decided to add the option 'doubt' to the answer options 'yes' and 'no'. This way, the question would be perceived as less definite by professionals |
| **ERASE signalling questions:** |  |  |  |
|  | SQ1 | No modifications | Respondents noted the word "appropriate" to be subjective and perceived as judgmental. These respondents would like to see an alternative wording. Possible option after the evaluation was to add an additional pop-up 'Be aware of your own frameworks'. This is a nice option, but not concrete enough. The project group looked again for alternatives, but did not find them. The wording remained 'appropriate'. |
|  | SQ2 | No modifications | Respondents found this question very relevant, explanation under the pop-up was clear. No improvement suggestions were made and therefore no adjustment was needed. |
|  | SQ3 | No modifications | It was mentioned that an unexplained delay in seeking help was relevant, but sometimes difficult for respondents. The project group looked again at the question and judged it to be sufficiently clear. There seemed to be a knowledge deficit among the professionals. |
|  | SQ4 | No modifications | The project group discussed the tension between clarifying and not too long a questionnaire/texts in the EMR (workability). The suggestion of a respondent to add a anatomic drawing with locations suspected of inflicted injury as an example, found support in the project group. |
|  | SQ5 | No modifications | Respondents found this question relevant and had no suggestions for improvement. This question remained unchanged. |
|  | SQ6 | No modifications | Respondents did not find the question, "Are there other signs?" supportive or relevant in identifying elder abuse. During the evaluation, it turned out that it was not sufficiently clear to the employees why this question had been added. The project group had added this question precisely so as not to make the questionnaire too long or exhaustive and not to make it look like cookbook medicine. The project group decided after the evaluation not to add more examples under this question (there were pop-up examples already present), because of the earlier argumentation (supportive, but not exhaustive, keep it short but to the point). |
